# AMSunda: A novel dataset for Sundanese information retrieval

**DOI:** 10.1016/j.dib.2025.111796

**Published:** 2025-06-18

**Authors:** Aries Maesya, Yulyani Arifin, Amalia Zahra, Widodo Budiharto

**Affiliations:** a Computer Science Department, BINUS Graduate Program—Doctor of Computer Science Program, Bina Nusantara University, Jakarta 11480, Indonesia; b Computer Science Department, School of Computer Science, Bina Nusantara University, Jakarta 11480, Indonesia

**Keywords:** Sundanese language, Sundanese dataset, Information retrieval, Text embedding, Natural language processing

## Abstract

Information Retrieval is crucial in many areas, including Search Engines, Information Systems, and Databases. As an indigenous language, the Sundanese corpus from West Java in Indonesia suffers from limited data availability, especially for Information Retrieval tasks. Previous efforts to build the Sundanese dataset mainly focused on text classification and generation, leaving information retrieval tasks underexplored. To address this gap, we named the AMSunda dataset. The AMSunda dataset was introduced as the first resource designed explicitly for fine-tuning and evaluating embedding models in the Sundanese language. AMSunda dataset consists of two dataset types: (1) triplet data containing a query passage, a positive, and a negative response aimed for fine-tuning embedding models, and (2) BEIR-compatible data structured for evaluating embedding models on retrieval tasks. The dataset consists of 1499 documents generated using GPT-4o-mini LLM, resulting in 7492 triplet passages and 7491 BEIR-format queries. This dataset enables further development of Sundanese-focused models in Information Retrieval.

Specifications Table

**Table:** 

Subject	Computer Science
Specific subject area	Natural Language Processing (NLP) with a focus on the creation and evaluation of Information Retrieval systems
Type of data	Benchmarking Information Retrieval (BEIR) dataset format for evaluating Information Retrieval systems and passage triplets for training embedding models with contrastive learning in JSON lines format (JSON)
Data collection	Collected Sundanese texts from various printed and online literature, including dictionaries, books, magazines, and online sources.
Data source location	Institution: Bina Nusantara University City/Town/Region: Jakarta Country: Indonesia
Data accessibility	The dataset in JSON lines format (JSON) file is freely available for public use and can be accessed at https://doi.org/10.5281/zenodo.15494944. Repository name: Zenodo DOI: 10.5281/zenodo.15494944
Related research article	None

## 1 Value of the Data

• Several datasets have been developed for fine-tuning and evaluating Information Retrieval systems, such as Microsoft Machine Reading Comprehension (MS-MARCO) [1] and Benchmarking Information Retrieval (BEIR) [2]. However, these datasets primarily offer content in English. Although recent initiatives in the Indonesian NLP community, such as NusaCrowd, have introduced various Sundanese language datasets, none have been created explicitly for Information Retrieval tasks [3].
• This dataset is the first resource curated explicitly for Information Retrieval in the Sundanese language. It was manually curated from various sources and verified by experts.
• This dataset consists of triplet (query passage, positive, and negative responses) data for training and fine-tuning an embedding model for semantic search and a BEIR-compatible data format for evaluating an information retrieval system. This structure ensures compatibility with widely used open-source NLP ecosystems for future research.
• In addition to Information Retrieval tasks, this dataset can be leveraged for a broader range of NLP applications, including Question Answering, Text Summarization, and Multilingual Transfer Learning. The availability of aligned Sundanese–English text further supports cross-lingual retrieval research and contributes to the development of multilingual NLP systems, particularly for low-resource languages.
• The structure of the dataset ensures compatibility with widely adopted open-source NLP ecosystems, facilitating reproducibility and promoting further research in regional language technologies.
• To demonstrate the initial utility of the dataset, several benchmark retrieval models such as TF-IDF and Transformer-based embedding models (e.g., MiniLM and DistilBERT) were evaluated. The results from these baseline experiments serve as reference points for future research and validate the dataset’s applicability for both traditional and neural Information Retrieval approaches.

## 2 Background

Information Retrieval (IR) has progressed rapidly with the rise of Machine Learning and Natural Language Processing (NLP) techniques [4,5]. However, these advancements have concentrated mainly on major languages such as English, Mandarin, and Spanish, while low-resource languages like Sundanese remain underrepresented. Sundanese, spoken by millions of people primarily in West Java, Indonesia, is a linguistically rich language with high cultural value [6]. Yet presents unique challenges and opportunities for IR research.

The development of effective IR systems for Sundanese faces several obstacles, including the lack of high-quality annotated datasets, substantial linguistic variation, and the limited available computational resources tailored to the language. Sundanese exhibits syntactic, semantic, and morphological characteristics that differ significantly from high-resource languages [7], requiring specialized text processing and model fine-tuning approaches.

Recent NLP research for low-resource languages emphasizes creating domain-specific and linguistically diverse datasets to support fine-tuning and evaluation processes [3]. Such datasets are critical for adapting pre-trained language models, such as BERT, GPT, or multilingual embeddings, to specific linguistic and cultural contexts [8]. For Sundanese, these resources are crucial to bridging the performance gap in applications like search engines [9].

No publicly available Sundanese language dataset exists for information retrieval tasks. [3]. The most recent initiatives to document Indonesian language resources, including Sundanese language datasets, primarily focus on text classification, generation, and translation [[10], [11], [12]]. This research introduces a novel Sundanese language dataset for fine-tuning and evaluating embedding models for information retrieval.

## 3 Data Description

This dataset comprises two dataset subsets: (1) a dataset for fine-tuning an embedding model based on the triplet dataset format and (2) a dataset for evaluating an information retrieval system based on the BEIR dataset format.

**Fig. 1:**
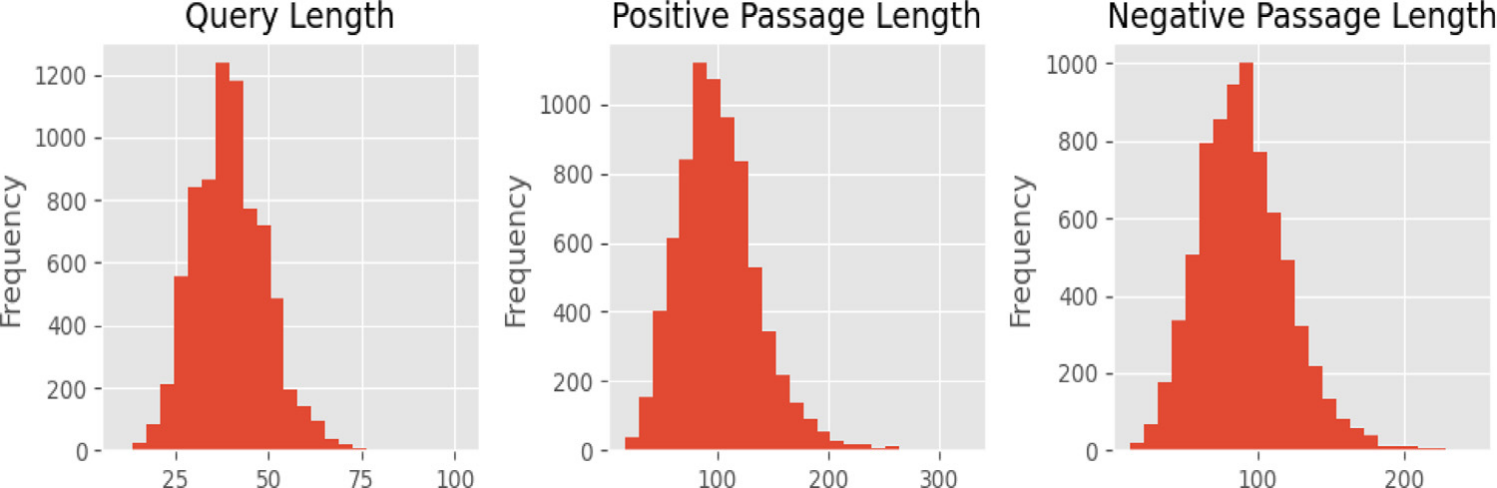
Distribution of passage lengths in the fine-tuning passage dataset.

The fine-tuning subset consists of three attributes: the query (or anchor texts), positive pairs, and negative pairs. This model includes 7492 datasets. The queries generally contain fewer than 100 characters (Fig. 1). Meanwhile, the positive and negative pairs may contain up to 350 characters. Table 1 shows a detailed list of the attributes included in the dataset.

**Table 1:** Dataset column description for fine-tuning the embedding model.

File Name	Attribute Name	Description
triplet.jsonl	Query	The given query to search for or the anchor text
	Positive	Relevant answer to the given query
	Negative	Irrelevant answer to the given query

To facilitate a clearer understanding of the dataset structure, Table 2 presents a sample row from the triplet file. Each triplet consists of a query written in Sundanese, a semantically relevant document (positive), and a non-relevant document (negative). This structure is designed to support ranking model training using contrastive learning approaches. English translations are included to enhance clarity and ensure accessibility for a broader research audience.

**Table 2:** Sample row from the triplet file.

Query	Positive	Negative

Sundanese: Kumaha cara ngahontal kahayang dina kahirupan? English: How to achieve desires in life?	Sundanese: Kahiji, urang kedah sabar sareng henteu janten kaganggu ku hasil nu henteu langsung katingali. English: First, we must be patient and not become complacent if results are not immediately visible.	Sundanese: Abdi ngadangu seueur warta ngeunaan jalma anu hasil tanpa usaha. English: I have heard a lot of news about successful people without effort.

**Fig. 2:**
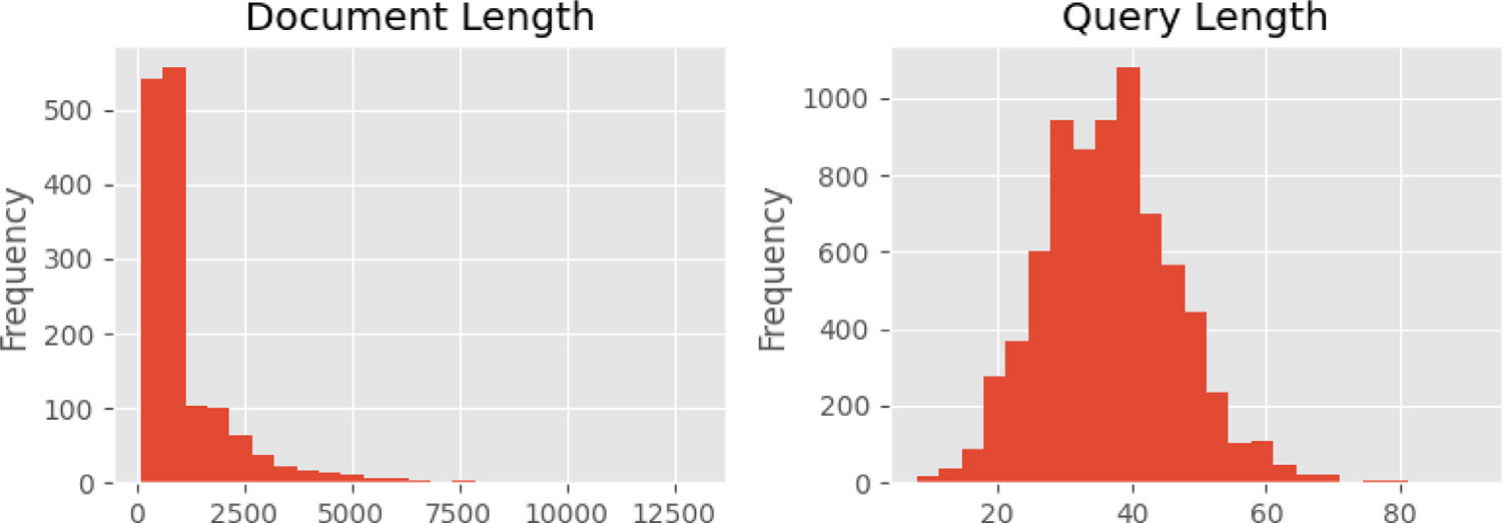
Distribution of passage lengths in evaluating the dataset corpus and query character.

The evaluation dataset consists of 1499 rows of documents, etc., accompanied by five queries, resulting in 7910 queries. The dataset is divided into three different BEIR-format files: corpus, queries, and qrels files, as described in Table 3. The corpus primarily contains short paragraphs with a few longer documents (Fig. 2). The queries, however, contain less than 100 characters, and the most common query length is approximately 37 characters.

**Table 3:** Dataset column description for evaluating information retrieval.

File Name	Attribute Name	Description
corpus.jsonl	document_id	Unique identifier for a given document
	Title	Document title
	Content	Document content
query.jsonl	query_id	Unique identifier for a given user search query
	Content	Short, user-generated search query
queries.jsonl	query_id	Unique identifier for a user query in the query.jsonl file
	document_id	Unique identifier for a relevant document in the corpus. The JSONL file is given the query_id

Table 4 illustrates a sample row from the queries file. Each entry in this file contains a unique query ID, a query written in Sundanese, and its corresponding English translation. These queries are utilized for both training and evaluation purposes, particularly in semantic retrieval tasks.

**Table 4:** Sample row from the queries file.

Document_id	Text	Text_English
0f0e9a58-c996-4926-9e6b-8ae1c646fe34	Naon anu dipikaharti ku naraka dina kahirupan?	What is understood by hell in life?

Each entry represents a relevance judgment that links a query ID to a corresponding document ID from the corpus, accompanied by a binary relevance score. The qrels file follows the standard format used in BEIR benchmarks and is crucial for evaluating the effectiveness of information retrieval models. A sample row from the qrels file as described in Table 5.

**Table 5:** Sample row from the qrels file.

Query_id	Corpus_id	Relevance
0f0e9a58-c996-4926-9e6b-8ae1c646fe34	c51ddd60-adc7-4b95-b09d-3c4865ba2aaf	1

Table 5 follows the BEIR qrels format. Each row indicates the relevance score between a query and a document, where 1 denotes relevance and 0 denotes non-relevance. These labels serve as ground truth for evaluating the performance of retrieval models.

**Fig. 3:**
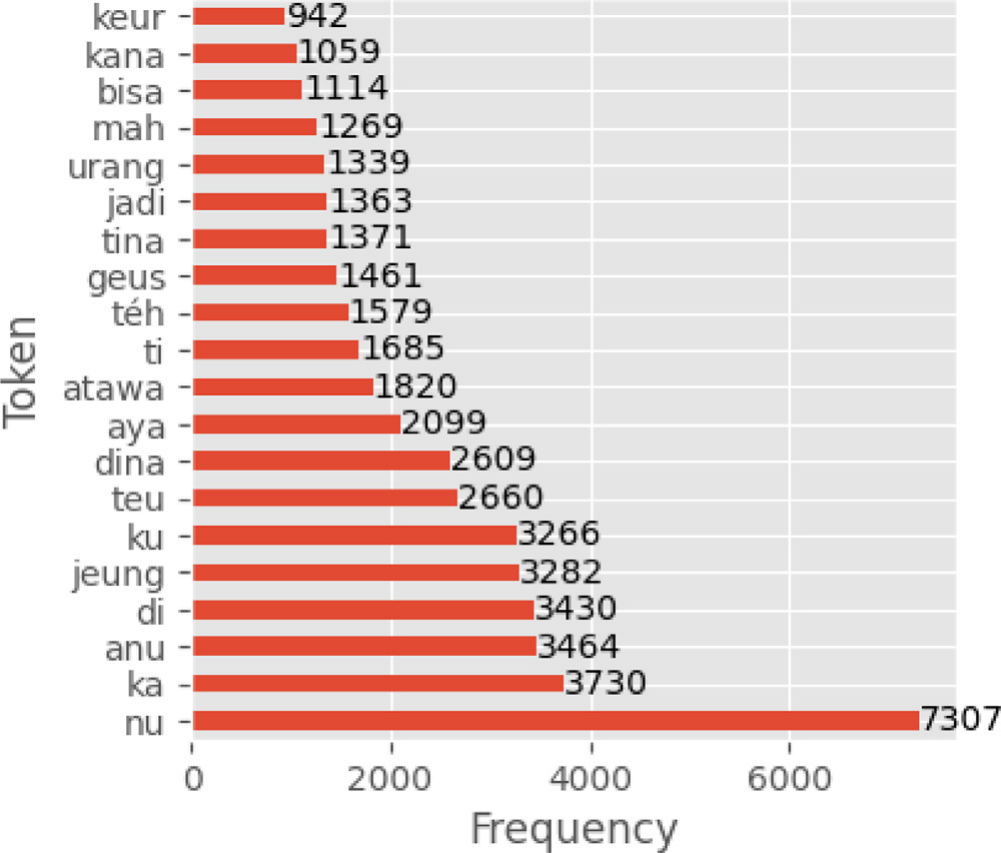
Top 20 most common tokens in the evaluation dataset corpus.

The distribution of tokens in the corpus shows a high number of stop words such as “nu,” “ka,” and “anu” (Fig. 3).

## 4 Experimental Design, Materials, and Methods

**Fig. 4:**
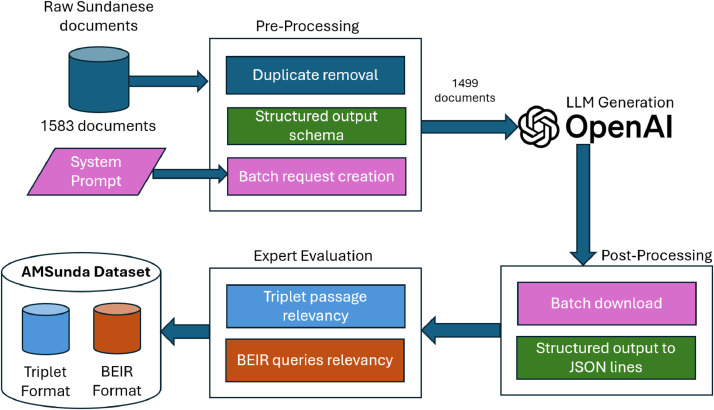
Activity flow of novelty dataset for Sundanese information retrieval dataset development (AMSunda Dataset).

The serial process through three main steps was conducted to produce a novelty Sundanese dataset for information retrieval (AMSunda Dataset) dataset development, as presented in Fig. 4. The following steps were undertaken in combination: manual data curation, review, experimental design, and automated dataset generation using large language models. Overall, this study is divided into three stages: data curation, data generation, and validation by a panel of experts.

### 4.1 Raw data collection

Collected Sundanese texts from various literature sources, including dictionaries [13], textbooks, magazines, and online sources. The collected texts were stored in a private online database for further processing. In total, 1583 documents form the basis for the synthetic dataset generation process. Therefore, it must be cleaned and structured before being used effectively for language model processing and dataset creation.

### 4.2 Pre-processing

In the pre-processing stage, the raw Sundanese documents are cleaned and prepared for input into the language model. This stage consists of three main steps:

• Duplicate Removal: Duplicate entries in the dataset are identified and removed to ensure the uniqueness and quality of the content. This step reduces noise and prevents redundancy in the final dataset.
• Structured Output Schema: This defines a consistent structure for the output data. This schema ensures that the language model produces results in a format that is easy to process and analyze later.
• Batch Request Creation: Organizes documents and system prompts into batches for LLM processing.

After pre-processing, the dataset is reduced from 1583 to 1499 documents, and it is now ready to be passed on to the language model for generation.

### 4.3 Dataset generation using LLM

In this stage, the 1499 pre-processed Sundanese documents are sent to a Large Language Model (LLM) developed by OpenAI. The model receives each document, a system prompt, and the defined output schema to guide the generation process. The dataset generation process is divided into two major phases: (1) the fine-tuning dataset in triplet format (query, positive, negative) and (2) the information retrieval evaluation dataset in BEIR format. The GPT-4o Mini large language model (LLM) [14]. Generated these datasets using a zero-shot prompting approach. The following system prompt guided the dataset generation process.

System prompt to generate the BEIR dataset:

**Table:** 

Generate five short search query-answer pairs based on the provided document. Each query should resemble a natural search input like those entered into Google or other search engines.
- Must write both the query and the answer in Sundanese.
- Ensure that each response is concise, accurate, and directly relevant to the content of the source document.

System prompt to generate the triplet dataset:

**Table:** 

Create an MS-MARCO triplet dataset from the provided document. Each triplet consists of:
1. Query: A short, natural search query based on the document's content.
2. Positive Passage: A passage taken from the document directly answers the query.
3. Negative Passage: A passage from the document that does not answer the query but remains topically related.
Requirements:
- Generate exactly five triplets.
- Write all elements (query, positive passage, negative passage) in Sundanese.
- Ensure the query and passages are concise and coherent.

The data generation process was carried out using the OpenAI Platform Batch API [15]. To call the LLM generation function and minimize costs efficiently. It took approximately 4 h to complete. The generated data was then processed using Python and the Pandas library [16]. To reformat the dataset into the appropriate triplet and BEIR format.

### 4.4 Post-processing

After the Large Language Model (LLM) generates structured outputs, the Post-Processing stage organizes and formats the results for further use. This stage includes two key steps:

• Batch Download: The generated outputs are collected in batches.
• Structured Output to JSON Lines: The structured outputs are converted into the JSON Lines format.

### 4.5 Expert evaluation

Because of the extensive data in the dataset, we employed a random sample for the review process. Selected several samples from each dataset subset for evaluation. The review process was conducted through interviews with a panel of experts. Assessed the validity of the synthetic data, and expert feedback was recorded to filter out the data with low-quality content. The evaluation focuses on two main aspects:

• Triplet Passage Relevancy: Experts evaluate if the three passages grouped (triplets) are relevant to each other. This is important for training models to understand and retrieve relevant documents.
• BEIR Queries Relevancy: Assesses how well the outputs respond to retrieval queries based on the BEIR format.

Extended Description of Triplet Generation and Expert Validation:

We employed a structured prompting strategy using the GPT-4o-mini language model to generate high-quality triplet data. Each selected document was input into the model along with a system prompt specifically designed to extract five distinct triplets. Each triplet consisted of a natural-language query, a relevant passage (positive), and an irrelevant yet topically related passage (negative). The relevance of the positive passage was determined by its direct response to the query within the context of the source document, while the negative passage was selected to be semantically unrelated but still derived from the same document to maintain contextual consistency.

Following automatic generation, a stratified random sampling approach was applied to select approximately 10 % of the triplets for human validation. Three independent native Sundanese language experts were tasked with evaluating the selected triplets. They assessed each component for linguistic clarity, relevance, and coherence. Any triplet containing ambiguous queries, incorrectly matched responses, or poor language construction was flagged and excluded from the final dataset. The experts’ feedback also informed revisions to the prompting schema and served as a quality assurance step to ensure that the dataset is both linguistically accurate and suitable for fine-tuning semantic retrieval models.

### 4.6 Final output – AMSunda dataset

After completing all the previous stages, including data cleaning, LLM generation, and expert evaluation, the final result is the AMSunda Dataset, a high-quality resource for research and applications in Sundanese information retrieval. The dataset is provided in two formats, Triplet Format and BEIR Format, to support different use cases.

## Limitations

Not applicable.

## Ethics Statement

This dataset was developed using Sundanese texts collected from various printed and online sources, including dictionaries, textbooks, magazines, and public web content. All data used in the dataset were publicly available or under fair use provisions for research and educational purposes. Where applicable, sources such as dictionaries were cited correctly, and no proprietary or copyrighted material was included without the author’s permission. The dataset contains no personal or sensitive information and was reviewed by experts to ensure ethical data usage.

## Credit Author Statement

**Aries Maesya**: Conceptualization, Methodology, Data Curation, Software, Original draft preparation, Visualization. **Yulyani Arifin**: Supervision, Validation, Investigation. **Amalia Zahra**: Supervise, validate, write, review, and edit. **Widodo Budiharto**: Supervision, Validation, Investigation. All authors read and approved the manuscript.

## Data Availability

zenodoAMSunda: A Novel Dataset for Sundanese Information Retrieval (Original data).

## References

[1] P. Bajaj, D. Campos, N. Craswell, et al., “MS MARCO: A human-generated machine reading comprehension dataset,” arXiv preprint, arXiv:1611.09268, 2016. 10.48550/arXiv.1611.09268.

[2] Thakur N., Reimers N., Rücklé A., Srivastava A., Gurevych I. (2021). Proceedings of the Neural Information Processing Systems Track on Datasets and Benchmarks.

[3] Cahyawijaya S., Rogers A., Boyd-Graber J., Okazaki N. (2023). Findings of the Association for Computational Linguistics: ACL 2023.

[4] Akkalyoncu Yilmaz Z., Wang S., Yang W., Zhang H., Lin J., Padó S., Huang R. (2019). Proceedings of the 2019 Conference on Empirical Methods in Natural Language Processing and the 9th International Joint Conference on Natural Language Processing (EMNLP-IJCNLP): System Demonstrations.

[5] Yang Y., Qiao Y., Shao J., Yan X., Yang T. (2022). *Proceedings of the Fifteenth ACM International Conference on Web Search and Data Mining*, in WSDM ’22.

[6] Kurniawati W., Akhadiah S., Emzir (2020). Sundanese language maintenance in Cianjur city (Ethnographic Research). Int. J. Sci. Res. Manag. (IJSRM).

[7] Alhammad R. (2023). The phonology, morphology, and syntax of Sundanese. Forum Linguist. Stud..

[8] N. Reimers and I. Gurevych, “Sentence-BERT: sentence embeddings using siamese BERT-networks,” arXiv preprint arXiv:1908.10084, 2019. doi:10.48550/arXiv.1908.10084.

[9] Heristian S., Kautsar H.A.A., Sayfulloh A. (2019). Rancang Bangun information retrieval system (IRS) Kamus Bahasa-Sunda. Com Dengan metode vector space model (VSM). JITK (J. Penget. dan Teknol. Komput.).

[10] S. Cahyawijaya et al., “IndoNLG: benchmark and resources for evaluating indonesian natural language generation,” in *Proceedings of the 2021 Conference on Empirical Methods in Natural Language Processing*, M.-F. Moens, X. Huang, L. Specia, and S.W. Yih, Eds., Online and Punta Cana, Dominican Republic: Association for Computational Linguistics, 2021, pp. 8875–8898. doi: 10.18653/v1/2021.emnlp-main.699.

[11] A. Conneau et al., “Unsupervised cross-lingual representation learning at scale,” in *Proceedings of the 58th Annual Meeting of the Association for Computational Linguistics*, D. Jurafsky, J. Chai, N. Schluter, and J. Tetreault, Eds., Online: Association for Computational Linguistics, 2020, pp. 8440–8451. doi: 10.18653/v1/2020.acl-main.747.

[12] B.D. Trisedya and D. Inastra, "Creating indonesian-javanese parallel corpora using Wikipedia articles," in *2014 International Conference on Advanced Computer Science and Information Systems*, 2014, pp. 239–245. doi: 10.1109/ICACSIS.2014.7065828.

[13] Danadibrata R.A. (2006).

[14] OpenAI, GPT-4 Technical Report, arXiv preprint arXiv:2303.08774, 2023. Available: https://arxiv.org/abs/2303.08774. doi:10.48550/arXiv.2303.08774.

[15] OpenAI, “Batch API,” OpenAI Platform. Accessed: Jan. 20, 2025. [Online]. Available: https://platform.openai.com

[16] (2020). The pandas development team, *pandas-dev/pandas: pandas*. Zenodo.

